# Heterogeneity and Diversity of Striatal GABAergic Interneurons: Update 2018

**DOI:** 10.3389/fnana.2018.00091

**Published:** 2018-11-08

**Authors:** James M. Tepper, Tibor Koós, Osvaldo Ibanez-Sandoval, Fatuel Tecuapetla, Thomas W. Faust, Maxime Assous

**Affiliations:** Center For Molecular and Behavioral Neuroscience, Rutgers University, Newark, NJ, United States

**Keywords:** interneurons, GABA, striatum, neurophysiology, neuroanatomy

## Abstract

Our original review, “Heterogeneity and Diversity of Striatal GABAergic Interneurons,” to which this is an invited update, was published in December, 2010 in Frontiers is Neuroanatomy. In that article, we reviewed several decades’ worth of anatomical and electrophysiological data on striatal parvalbumin (PV)-, neuropeptide Y (NPY)- and calretinin(CR)-expressing GABAergic interneurons from many laboratories including our own. In addition, we reported on a recently discovered novel tyrosine hydroxylase (TH) expressing GABAergic interneuron class first revealed in transgenic TH EGFP reporter mouse line. In this review, we report on further advances in the understanding of the functional properties of previously reported striatal GABAergic interneurons and their synaptic connections. With the application of new transgenic fluorescent reporter and Cre-driver/reporter lines, plus optogenetic, chemogenetic and viral transduction methods, several additional subtypes of novel striatal GABAergic interneurons have been discovered, as well as the synaptic networks in which they are embedded. These findings make it clear that previous hypotheses in which striatal GABAergic interneurons modulate and/or control the firing of spiny neurons principally by simple feedforward and/or feedback inhibition are at best incomplete. A more accurate picture is one in which there are highly selective and specific afferent inputs, synaptic connections between different interneuron subtypes and spiny neurons and among different GABAergic interneurons that result in the formation of functional networks and ensembles of spiny neurons.

## Introduction

When we sat down to write the first iteration of this review (*Heterogeneity and Diversity of Striatal GABAergic Interneurons*, Tepper et al., 2010), we were aware of four classes of striatal GABAergic interneurons. These comprised the parvalbumin (PV)- expressing fast spiking interneurons (FSI), the neuropeptide Y (NPY)/somatostatin (SOM)/nitric oxide synthase (NOS) (P)LTS interneurons, calretinin (CR) expressing interneurons, and a newly discovered group of tyrosine hydroxylase (TH)-expressing interneurons subsequently termed THINs.

We reviewed what was then the current state of knowledge of the electrophysiological, morphological and pharmacological properties of these interneurons, along with their synaptic connections. Since then, there has been considerable progress made in extending these observations, and, largely due to the availability of new lines of BAC transgenic mice comprising selective Cre- drivers and fluorescent reporters with specific interneuronal types expressing GFP or EGFP, several additional striatal GABAergic interneuron subtypes have been discovered. In this updated review, written especially for the 10th anniversary of the Frontiers journals, we will not revise the original review, and repeat what was written before, but rather focus on what is new since 2010. For basic information on fast spiking interneurons (FSIs), CR, low threshold spike (LTS) and THINs, refer to the original (Tepper et al., 2010; see also Tepper et al., 2004; Fino and Venance, 2011). This review will begin with what is currently known about the newer “novel” GABAergic interneurons, and in the second part, we will update the descriptions of the “older” interneurons with recent findings.

## Novel GABAergic Interneurons

### Neurogliaform Interneurons

Striatal NPY-expressing interneurons were originally considered to consist of one class of GABAergic interneurons. These cells were also known to express SOM and NOS (Vincent and Johansson, 1983; Vincent et al., 1983), and to display a unique electrophysiological profile including a high input impedance (>600 MΩ), relatively long duration action potentials, a prominent AHP, low threshold Ca^2+^ spikes (LTS) and a prolonged plateau potential leading them to be termed PLTS (see “LTS Interneurons” section below for explanation of terminology) interneurons (Kawaguchi, 1993; Kawaguchi et al., 1995; Centonze et al., 2002). When the NPY-GFP transgenic mouse became available, it became clear that there were not one but two very different NPY-expressing neurons in striatum. About 75% of the striatal NPY interneurons labeled in this mouse were the previously described P/LTS interneurons (see below), but 25% exhibited a completely different set of electrophysiological properties and a markedly different morphology (Ibáñez-Sandoval et al., 2011; Beatty et al., 2012; English et al., 2012).

#### Neurocytology

**Figure 1:**
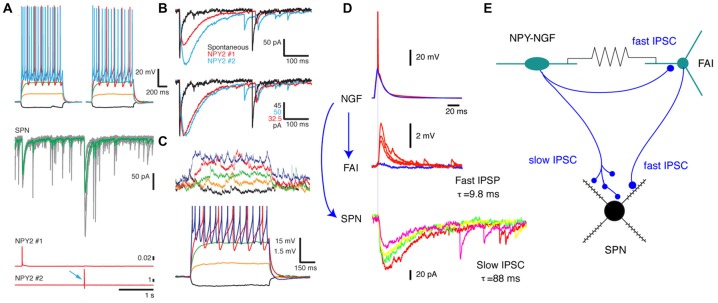
Synaptic connections and electrotonic coupling of neurogliaform (NGF) interneurons. **(A)** Simultaneous triple recording from two neuropeptide Y (NPY)-NGF interneurons (top panels, whole cell current voltage series) and an spiny projection neuron (SPN) (middle, voltage clamp). Spikes elicited in the two NGF interneurons (botttom, red traces) evoke IPSCs in the SPN (middle, arrows; individual traces, gray; average, red). **(B)** Overlay of the average IPSCs elicited in the SPN (top) by the two interneurons (bottom panel: peak-scaled IPSCs). Note the nearly identical, characteristic slow time courses of the GABA_Aslow_ IPSCs (blue and red). A regular, fast spontaneous GABA_A_ IPSC is shown on the same scale (black) for comparison to illustrate the kinetic difference between the responses. **(C)** Electrotonic coupling between the same two NPY-NGF interneurons shown by membrane potential deflections in one neuron (top traces) induced by hyperpolarizing (black) and depolarizing potentials in the other neuron (bottom traces). Scale bar are the same for both NGFs. **(D,E)** Different triple whole cell recording illustrates complex interconnections of an NGF, fast adapting interneuron (FAI) and SPN. **(D)** The NGF (top) synapses onto both the FAI (middle) and the SPN (bottom). Note that while the synapse onto the SPN elicits a GABA_Aslow_ IPSC, the synapse onto the FAI, arising from the same axon exhibits normal fast GABA_A_ kinetics. **(E)** Schematics illustrating the connections of the three neurons. Panels **(A–C)** adapted from English et al. (2012).

When striatal sections of BAC transgenic mice that expressed the humanized Renilla green fluorescent protein under the control of the mouse NPY promoter (NPY-GFP mice; Jackson Laboratory) were examined two distinct cell types were readily apparent, both of which were immunopositive for NPY (Ibáñez-Sandoval et al., 2011). About 25% of the immunostained neurons were significantly brighter, and had more complex dendritic arborizations, than the other 75% of the fluorescent neurons (Figures 1A,B and Table 1). Additional immunofluorescence studies revealed that the more common, less bright cell type also expressed SOM and NOS, as had been shown previously to be the case for cells identified as LTS interneurons (Kawaguchi, 1993). In contrast, the brighter neurons did not express either peptides (Ibáñez-Sandoval et al., 2011).

**Table 1:** Main anatomical and electrophysiological characteristics of striatal GABAergic interneurons.

	Markers	Morphology	Input Res	RMP	Spont Act	Connectivity with SPNs	Connectivity with other interneurons
**FSI**	PV	At least two subtypes, medium to large soma, 5–8 principal dendrites higher order varicose dendrites forming 200–300 μm diameter field, very dense, highly branched axon, 1.5–2 times wider than the dendritic field	50–100 MΩ	~−80 mV	No	High ~80%	FSI (electrotonic coupling), NGF synaptic
**LTS**	NPY/NOS/SOM	Medium sized soma, 3–5 aspiny dendrites, with little branching, very long sparsely branching axon extending 600–1000 μm from soma forming infrequent bouquets with varicosities	>600 MΩ	~−56 mV	Yes	low ~20%	CINs
**CR**	CR	Multiple types with variable morphology, small to medium sized soma, some with very few spiny dendrites, other multipolar with smooth aspiny dendrites	?	?	?	?	?
**THIN**	TH	One(Type I) principal and three less frequent subtypes, all with medium sized somata, Type I emits 2–4 primary dendrites, higher order dendrites sparsely spinous, modest, dense, highly branched axon studed with varicosities	350–1,500 MΩ	~−50 mV	Yes	low ~20% Reciprocal	LTS, CINs
**NFG**	NPY	Medium sized soma, 5–9 aspiny primary dendrites, very dense and highly compact higher order dendritic field, very dense axonal arborization extending throughout and beyond dendritic field	~140 MΩ	~−85 mV	No	High ~80%	NGF (electrotonic coupling), FAI, CINs
**FAI**	Htr3a	Medium sized soma, 3–5 aspiny varicose dendrites, relatively dense axonal field	~362 MΩ	~−65 mV	No	High ~50%	?
**SABI**	Htr3a	Medium sized soma, 3–5 aspiny varicose dendrites, sparsely spiny, sparse axonal arborization	>600 MΩ	~−50 mV	Yes	No (~4%)	?

**Figure 2:**
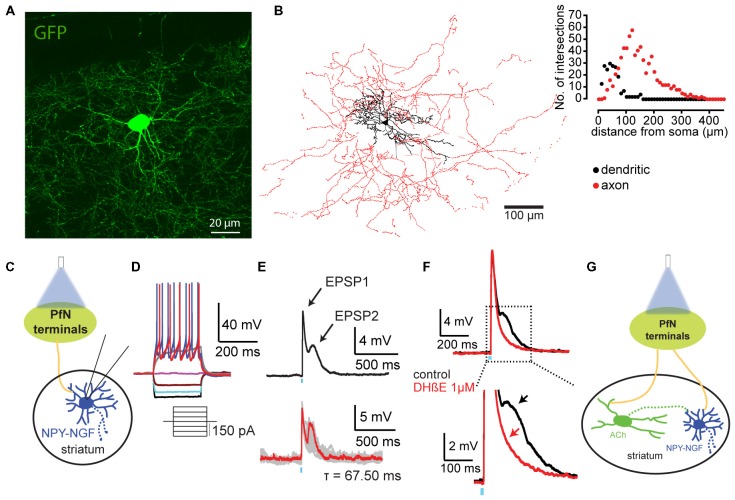
Thalamic innervation of NGF interneurons. **(A)** Total projection of a confocal stack of an NGF interneuron (expressing GFP, NPY-GFP mice; **B)**. 3D reconstruction of an NPY-NGF interneuron filled with biocytin. The soma and dendritic fields are represented in black and the axon in red in the reconstructed image. **(C)** Cartoon illustrating the experimental design where we recorded striatal NGF interneurons response to PfN optogenetic stimulation. **(D)** Typical voltage response of a NGF interneuron to somatic current injection. **(E)** Optogenetic stimulation of PfN striatal terminals induces biphasic excitatory responses. **(F)** While the first EPSP (EPSP1) is glutamatergic the second one (EPSP2) is due to thalamic activation of striatal cholinergic interneuron (CIN; **G**) as shown by DHβE blockade. Panel **(B)** adapted from Ibáñez-Sandoval et al. (2011); panels **(C–G)** adapted from Assous et al. (2017).

Subsequent whole cell recording and biocytin filling revealed that this second population of neurons exhibited a completely different morphology than the LTS interneurons. Their somata were slightly smaller than the LTS neurons (12.6 ± 0/7 × 9.4 ± 0.6 μm, 15.6 ± 0.8 × 9.5 ± 0.5 μm, respectively), and both the dendritic and axonal arborizations were markedly different. Simple visual inspection of the neuronal reconstructions as well as Sholl plot analyses revealed that the somata of these neurons issues 5–9 primary dendrites, and the dendritic tree was far denser and more highly branched, as well as significantly more compact than that of the LTS interneuron, averaging 200 μm or less in diameter. The axonal arborization was also much denser, more highly branched and more compact than that of the LTS interneuron, and expressed prominent small round varicosities, presumably synaptic boutons along the length of most axonal segments. The axonal field was anisotropic and extended throughout and beyond the dendritic tree, exceeding 400 μm in diameter as shown in Figure 2B and Table 1 (Ibáñez-Sandoval et al., 2011). Partly on the basis of this cellular morphology, previously described in cortex and hippocampus (Karagiannis et al., 2009; Fuentealba et al., 2010; Armstrong et al., 2012) and partly on the basis of its neurophysiology (described below), we termed this second type of NPY interneuron the striatal neurogliaform (NGF) interneuron. Like the LTS interneuron, the dendrites of the NGF interneuron were very sparsely spiny.

#### Intrinsic Electrophysiological Properties

Striatal NGF interneurons exhibited many electrophysiological properties that were similar to those previously described for cortical, hippocampal and amygdala NPY-expressing NGF interneurons (e.g., Povysheva et al., 2007; Karayannis et al., 2010; Mańko et al., 2012). These include a low input impedance (74–260 MΩ), strong inward rectification, little spike frequency adaptation, and the absence of I_h_, LTS or plateau potentials (Ibáñez-Sandoval et al., 2011; English et al., 2012; Luo et al., 2013; Assous et al., 2017; Figure 2). NGF interneurons exhibit a very hyperpolarized resting membrane potential (RMP) in *ex vivo* recordings, and, like spiny projection neurons (SPNs), are not spontaneously active (Table 1). In fact, except for the very prominent deep and long lasting spike afterhyperpolarization (26 ± 1 mV; Ibáñez-Sandoval et al., 2011) that is their principal distinguishing characteristic, current voltage series of NGF interneurons in current clamp recordings bear a strong resemblance to that of SPNs, and could have been encountered previously and mistaken for SPNs upon superficial examination in the past.

Like NGF interneurons in other brain regions (Price et al., 2005; Simon et al., 2005), multiple striatal NGFs form an interneuronal network by virtue of their interconnection by monosynaptic electrotonic synapses (English et al., 2012; Assous and Tepper, 2018; Figures 1C,E and Table 1). In addition, NGFs are electrotonically connected to fast adapting interneurons (FAIs) and THINs by heterosynaptic electrotonic junctions (Assous et al., 2017).

#### Afferent Connectivity

Striatal NGF interneurons receive glutamatergic inputs from cortex. Interestingly, and in contrast to LTS interneurons (see below), with either electrical (Ibáñez-Sandoval et al., 2011) or optogenetic cortical stimulation (Assous et al., 2017), NGF interneurons respond mostly with subthreshold EPSP/Cs, and action potentials are rarely elicited. Striatal NGF interneurons also receive strong excitatory inputs from the parafascicular nucleus of the thalamus (PfN) that consist of mixed AMPA/NMDA EPSP/Cs that are often suprathreshold, driving single action potentials in response to brief single optogenetic activation of parafascicular terminals (Figures 2C–G; Assous et al., 2017). This differential response to cortical and thalamic inputs is a mirror image of the case with LTS neurons that will be described below in the Updates section (Assous and Tepper, 2018). NGF interneurons also exhibit IPSCs in response to optogenetic activation of PV-FSIs (Lee et al., 2017).

NGF interneurons express Type 2 nicotinic receptors that can be blocked by low concentrations of DHβE (Figures 2C–G; Ibáñez-Sandoval et al., 2011; English et al., 2012; Luo et al., 2013; Assous et al., 2017). In paired whole cell recordings these Type 2 nicotinic receptors are activated by monosynaptic inputs from striatal cholinergic interneurons (CINs) that lead to spiking in the NGFs (English et al., 2012). Brainstem cholinergic neurons have also recently been shown to project to striatum (Dautan et al., 2014), but it remains unclear if they might also contribute to nicotinic responses of NGF interneurons.

#### Efferent Connectivity

Like most other striatal GABAergic interneurons (see below), NGF interneurons synapse onto SPNs (Figures 1A,B,D,E; Table 1; Ibáñez-Sandoval et al., 2011; English et al., 2012). The connection probability in brain slices is extremely high, over 85%. Considering the very high likelihood that some presynaptic axons and/or postsynaptic dendrites are destroyed in 300 μm brain slices, similar to the case with FSIs, it is highly likely that most or all SPNs within the axonal arborization of an NGF interneuron receive synaptic input from one or more NGFs.

The NGF-evoked synaptic response is mediated by GABA_A_ receptors and is completely blocked by bicuculline. However, unlike that of all other striatal interneurons known to date, the NGF synapse elicits an IPSC/P with unusually slow kinetics, with a rise time around 10 ms and decay time over 120 ms, about 10 times slower (Figures 1B,D,E; Ibáñez-Sandoval et al., 2011; English et al., 2012; Assous et al., 2017).

This is similar to a GABA_Aslow_ current that has previously been described in cortex, hippocampus and amygdala (Banks et al., 1998; Banks and Pearce, 2000; Price et al., 2005; Fuentealba et al., 2008; Mańko et al., 2012). The slow kinetics are likely due to a combination of an extrasynaptic location of the receptor, lacking the typical ultrastructural synaptic morphology (e.g., Mańko et al., 2012) and the presence of the GABA_A_ β3 subunit (Capogna and Pearce, 2011; Luo et al., 2013). This GABA_Aslow_ synaptic response is an extremely powerful source of inhibition to the SPNs, not only because of its amplitude but also because of the extremely long duration and slow decay of the IPSC, and is responsible at least in part, for the inhibition of SPNs that follow optogenetic excitation of CINs (English et al., 2012; Faust et al., 2016). This circuit is active *in vivo* as well as in brain slices where optogenetic inhibition of CINs silences them. Upon release from the optogenetic inhibition, the CINs exhibit a rebound burst that leads to inhibition of firing of SPNs (English et al., 2012; but see also Zucca et al., 2018).

### HTR3a-Cre Targeted Interneurons

Within the cerebral cortex, neurons expressing PV, SOM, or the ionotropic serotonin receptor, 5HT3a, constitute almost the entire interneuronal population (Rudy et al., 2011). This prompted the creation of a BAC transgenic mouse line expressing Cre under the control of the 5HT3a (Htr3a) promoter (Gerfen et al., 2013). This mouse line has been extremely useful for the study of striatal neurocytology and has allowed for the discovery of novel interneurons that do not express previously described striatal GABAergic interneuronal markers (e.g., PV, SOM, NOS, TH), for which the physiology, morphology and connections of these targeted interneurons type have been at least initially described.

When we examined striatal sections from Htr3a-Cre mice locally injected with a fluorescently labeled, floxed AAV, we found multiple cell types labeled. The majority of the Htr3a-Cre transduced neurons coexpressed PV (~75% of the transduced neurons) or NPY (3.2%; Faust et al., 2015). The PV immunofluorescent neurons all proved with whole cell recording to be FSIs. Interestingly, all of the NPY immunofluorescent neurons exhibited the electrophysiological and morphological characteristics of NGF interneurons; i.e., NPY-LTS interneurons were not labeled in this preparation (Faust et al., 2015).

We also found that 2.3% of the labeled neurons expressed CR while none expressed NOS or SOM (consistent with the absence of neurons exhibiting the electrophysiological or morphological phenotypes of LTS interneurons) or TH. In total, 19.5% of the transduced neurons did not express any of the markers listed above. It was possible to divide a subset of these remaining neurons on the basis of subsequent electrophysiological and morphological experiments into two additional novel subtypes of striatal GABAergic interneurons: FAI (Faust et al., 2015) and spontaneously active bursty interneurons (SABIs; Assous et al., 2018).

### Fast-Adapting Interneuron

#### Neurocytology

**Figure 3:**
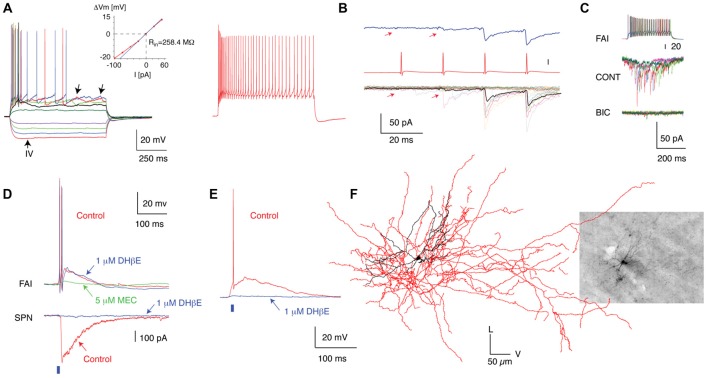
Anatomical, electrophysiological and circuit properties of FAI. **(A)** Membrane potential responses of a typical FAI to injected current pulses. Note the pronounced spike frequency adaptation and irregular membrane potential fluctuations (left panel, arrows). Inset shows the current–voltage relationship of this neuron. **(B)** IPSC trains in an SPN elicited by trains of presynaptic action potentials in a FAI. **(C)** IPSCs in SPNs are blocked by a GABA_A_ receptor antagonist (bicuculline, 10 μM). **(D)** Simultaneous recording from an FAI and an SPN. Optogenetic activation of cholinergic inputs (2-ms pulse of blue light, blue bar) elicited a large-amplitude IPSC in the SPN (bottom) and an EPSP giving rise to action potentials in the FAI (top). Note the diversity of pharmacological blockade in FAI in **(D,E)**. While application of DHbE (1 μM) block the IPSC in SPNs and the nicotinic EPSP in some FAI **(E)** it has no effect on other FAI (**D**; upper traces). In this case the nicotinic EPSP is blocked by MEC (5 μM). **(F)** 3D reconstruction of a FAI filled with biocytin after recording (Inset). The soma and dendritic fields are represented in black and the axon in red in the reconstructed image. Figure adapted from Faust et al. (2015).

FAIs were identified in the Htr3a-Cre transgenic mouse as relatively uncommon interneurons among the predominantly targeted FSIs based on their less intense fluorescence, smaller cell bodies, and thin dendrites under epifluorescence illumination (Faust et al., 2015). Based on a limited number of filled and reconstructed neurons, FAIs exhibited medium-sized, isotropic somata with 3–5 aspiny and varicose dendrites extending up to 150 μm from the cell body (Figure 3 and Table 1). Their axonal field overlapped and in some cases extended beyond the dendritic arbor. FAIs represent about 37% of the immunofluorescently uncharacterized Htr3a-cre transduced neurons (25/68 neurons) which would represent 7.02% of all the Htr3a targeted interneurons (Faust et al., 2015; Assous et al., 2018).

#### Intrinsic Electrophysiological Properties

Compared to most other striatal GABAergic interneurons, FAIs exhibited a relatively depolarized RMP (−66.2 mV, *n* = 25) and a relatively high input resistance (362.0 MΩ, *n* = 25). Suprathreshold depolarizations elicit a high initial firing rate followed by a pronounced spike-frequency adaptation, giving rise to their name. In some cases, FAIs displayed irregular membrane potential fluctuations following spike trains. Unlike THINs and SABIs (see below) that undergo depolarization block and complete spike failure during modest depolarizing current injections, FAIs increase their firing rate linearly to current injection pulses up to at least 250 pA. These intrinsic characteristics demonstrated some similarities to Type II and Type IV THINS, with key differences being the absence of spontaneous activity, plateau potentials, and rebound spiking from low threshold depolarizations in the FAI. These intrinsic properties, together with the absence of TH expression and the failure to observe these characteristic in any neurons labeled in slices from TH-EGFP or TH-Cre transgenics, served to characterize the FAI as a unique interneuron type.

#### Afferent Connections

FAIs receive a powerful nicotinic input from an as of yet unidentified source, likely to be striatal CINs but possibly partially or exclusively from cholinergic pedunculopontine axons innervating the striatum (Dautan et al., 2014). Optogenetic stimulation of local cholinergic axons evokes suprathreshold excitatory nicotinic responses in the majority of FAIs (10/13 neurons tested), consistent with their high input resistance and depolarized RMP. Interestingly, the nicotinic response was pharmacologically heterogeneous, with the majority of responses sensitive to low concentrations of the Type III nicotinic receptor antagonist, mecamylamine. However, a limited number of FAIs responses to cholinergic stimulation were reduced by the type II nicotinic receptor antagonist DHβE. The relative input from thalamic and cortical glutamatergic sources remains unknown.

#### Efferent Connections

In *ex vivo* paired recordings, FAIs exhibit a relatively high connectivity probability with nearby SPNs (50%, 11/22 pairs tested; Figure 3B and Table 1). The resulting IPSCs were sensitive to the GABA_A_ receptor antagonist, bicuculline. However, in marked contrast to all other GABAergic synapses thus far characterized within the striatal microcircuit, FAIs induced IPSCs in SPNs exhibited a notable synaptic facilitation. On average the IPSC amplitude increased by a factor >2 from the first to the third IPSC in response to a 50 Hz train of spikes in the presynaptic FAI. The facilitation was so marked that in some cases, the initial presynaptic FAI spike in a train failed to produce any response in the postsynaptic SPN while later spikes evoked larger and larger IPSCs (Figure 3). On average, the IPSC was relatively small (population average <20 pA), measured at the soma. But the location of FAI-SPN synapses is not known. Based on their high connection probability with SPNs, it remains likely that FAI-SPN synapse participates in the information processing or coding of SPNs, perhaps through inhibition of distal dendritic regions of SPNs.

### Spontaneously Active Bursty Interneurons

The second novel cell type labeled in Htr3a-Cre mice comprised a population of spontaneously active interneurons. In current clamp recordings, these interneurons shared enough similarities with the Type I THIN that initially we thought that they were Type I THINs. Both the Type I THIN and the novel neurons virally transduced in Ht3rA mice exhibit spontaneous activity, a relatively high input resistance, a sag in the voltage response to hyperpolarizing current injections, and most characteristically extreme spike frequency accommodation leading to depolarization block during modest depolarizing current injections.

However, as mentioned above, none of the Htr3a-Cre targeted interneurons expressed TH, which indicates that the spontaneously active interneuron targeted in the Htr3a-Cre mice is a novel subtype of striatal GABAergic interneuron. Because of its spontaneous activity and highly bursty firing in cell attached mode (see below), these cells have been termed SABIs (Assous et al., 2018).

#### Neurocytology

**Figure 4:**
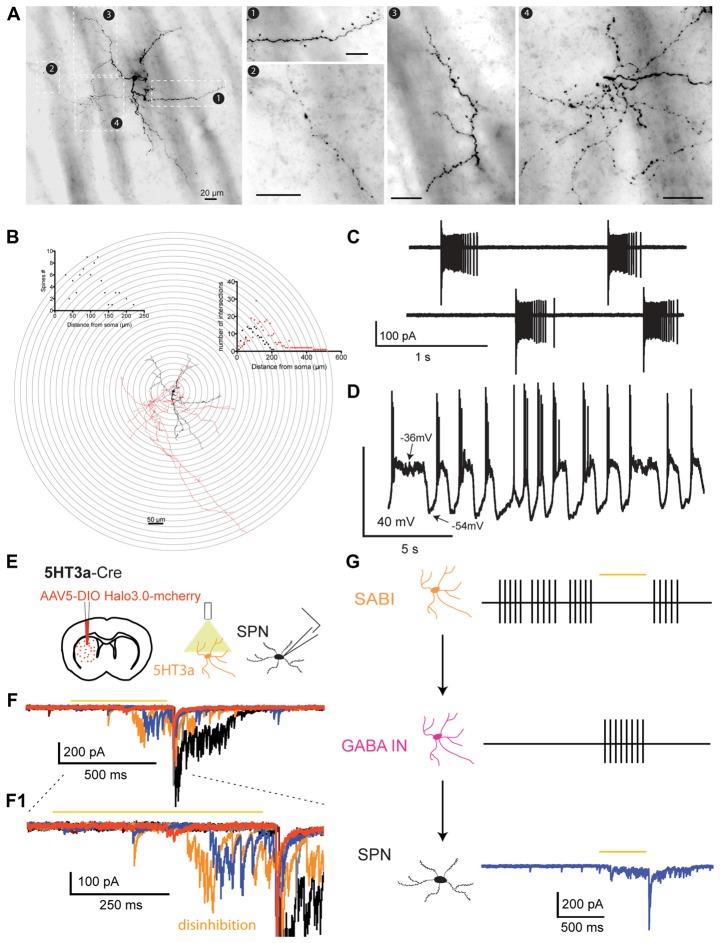
Anatomical and physiological properties of the spontaneously active bursty interneuron (SABI) and related circuit properties. **(A)** Neurocytology of typical electrophysiologically identified SABI interneurons labeled with biocytin after whole cell recording. SABIs emit several primary dendrites and secondary and higher-order dendrites are sparsely invested with dendritic spines (black arrows, box 1 and box 3). The axonal arborization was relatively sparse, exhibited prominent varicosities, and comprised small dense and tortuous fields near the soma (box 4), as well as sparse extended axons that extended well beyond the dendritic arborization (box 3). Scale bar value (20 μm) is the same for all panels. **(B)** 3D reconstruction and Sholl analysis of SABIs filled with biocytin after recording. The soma and dendritic fields are represented in black and the axon in red in the reconstructed image and the associated Sholl plot. **(C)** Representative cell-attached recordings of SABI exhibiting burst firing showing spike frequency adaptation, separated by periods of complete silence. **(D)** Spontaneous firing activity recorded in approximately half of SABI exhibit membrane potential fluctuations with action potential firing happening only during the beginning of the up state. **(E)** Schematic of the experimental paradigm. AAV5 Ef1a DIO HR3.0-EYFP was injected into the striatum of Htr3a-Cre mice and SPNs were recorded *ex vivo* using a cesium-based high-chloride internal solution (125 mM CsCl). **(F)** Representative examples of raw voltage-clamp data traces recordings in a SPN in the disinhibition protocol. Orange bar indicates the yellow light pulse. **(F1)** Expanded view of the disinhibitory IPSCs occurring during and immediately after the yellow light pulse. **(G)** Circuit diagram depicting the disinhibitory circuit hypothesized to mediate these responses. The Htr3a-Cre interneurons that are spontaneously active (i.e., the SABIs) are inhibited by halorhodopsin, which in turn disinhibits another, as yet unidentified population of interneuron(s) evoking these IPSC barrages in SPN. Figure adapted from Assous et al. (2018).

Electrophysiologically identified SABIs filled with biocytin after whole cell recording are medium-sized neurons emitting 3–5 primary dendrites that ramify into a relatively sparse anisotropic field approximately 300–400 μm in diameter. Secondary and higher order dendrites are only slightly varicose and are sparsely invested with dendritic spines. The axonal arborization was relatively sparse and generally comprised small, local fields near the soma as well as occasional sparse extended axons that extended well beyond the dendritic arborization (Figures 4A,B and Table 1). This likely has implications for the function of SABIs.

#### Intrinsic Electrophysiological Properties

Three populations of striatal interneurons have been previously described as being spontaneously active in slices: CINs, LTS interneurons and THINs. We recently showed that the SABI is an additional subtype of spontaneously active interneuron in the mouse striatum (Assous et al., 2018). In cell-attached recordings, the firing pattern of SABI is notably different from that of any of the other spontaneously active interneurons.

SABIs fire in highly irregular long bursts separated by very long pauses (up to several seconds). They fire nearly 100% of their spikes in highly adapting bursts of from about 25 to over 125 spikes fired at an average frequency of 100–300 Hz (Figure 4C).

In whole cell recordings, SABI exhibited a relatively high input resistance (>600 MΩ), and similarly to THINs, they exhibited dramatic depolarization inactivation when injected with moderate positive somatic current. SABI are also spontaneously active in whole cell mode where they exhibited two distinct firing patterns. About half of the neurons fired tonically at low rates and the other half exhibited large membrane potential fluctuations, similar to up and down states in described SPNs (Wilson, 1993; Wilson and Kawaguchi, 1996) with short bursts of action potentials riding on the beginning of the depolarization (Figure 4D).

#### Afferent Connectivity

The inputs to the SABI are still largely unknown. Our preliminary unpublished data show a strong glutamatergic innervation from the PfN and the cortex (Assous et al., unpublished). We also used double transgenic animals by crossing mice natively expressing ChR2 in cholinergic neurons (Choline Acetyltransferase, choline acetyltransferase (ChAT)-ChR2) with Htr3a-Cre mice to examine potential cholinergic inputs to the SABI. Optogenetic stimulation of cholinergic neurons evoke a strong depolarization of the SABI and action potential firing (Assous et al., unpublished). This excitatory response is nicotinic as it is blocked by mecamylamine but not DHβE or MLA, indicating that it is a Type III nicotinic receptor, distinct from the Type II nicotinic receptor (Albuquerque et al., 1995) expressed by NGF and most other striatal GABAergic interneurons.

#### Efferent Connectivity

Probably the most novel characteristic of the SABIs is that unlike all other striatal GABAergic interneurons identified to date, they do not significantly innervate SPNs. Using paired-recording in slices we found only 2 out of 45 connected pairs between nearby SABI and SPNs (4.4%; Table 1). Similarly, we observed the same very low connection probability in paired recordings when the SPNs were tested as presynaptic to SABIs (1 out of 24; 4.1%). These observations suggest that the principal synaptic targets of SABIs are other striatal interneurons.

Consistent with this suggestion, we found that optogenetic *inhibition* of Htr3a-cre targeted interneurons evokes IPSC barrages in SPNs (Assous et al., 2018; Figures 4E,F,F1). As the SABIs are the only known spontaneously active interneurons targeted in the Htr3a-cre mice, we hypothesized that the SABI are the ones responsible for the IPSCs barrages through the disinhibition of another distinct population of GABAergic interneuron that is not targeted in the Htr3a-cre mice (Figure 4G, see below). This population is not yet identified but might include THINs, LTS or another unknown population of GABAergic interneuron (see below) not transduced in the striatum of Htr3a mice.

These results suggest that the SABIs are the first example of an interneuron selective interneuron in the striatum. Such specialization in the interneuronal network suggests the existence of a hierarchical control of other striatal interneurons. The existence of an interneuron population specialized in synapsing onto other striatal interneurons could conceivably play an important role in the formation of ensembles of striatal SPNs.

In addition to the “novel” interneurons listed above, for which there is substantial electrophysiological and morphological characterization, there are several somewhat indirect lines of evidence that suggest the existence of at least two additional striatal GABAergic interneurons.

#### Recurrent Interneuron

In Sullivan et al. (2008), showed that GABAergic IPSCs could be elicited in CINs using extracellular electrical stimulation and more rarely, by the intracellular activation of single CINs. These IPSCs can be blocked by nonspecific GABA_A_ antagonists as well as well as those specific for β_2_-containing, Type II nicotinic receptor antagonists. These latter responses were termed recurrent IPSCs reflecting the reciprocal relationship between the source(s) of GABA release and the CINs. However, the GABAergic interneuron subtype mediating these responses does not appear to be any of the previously characterized Type II nicotinic receptors expressing interneurons (Assous et al., unpublished) and its identity, while unknown at present, may constitute yet another novel class of GABAergic interneurons.

#### Disinhibitory Interneuron

As mentioned in the “Spontaneously Active Bursty Interneurons” section above, whole cell recordings in Htr3a-Cre mice injected with halorhodopsion 3.0 showed that inhibition of spontaneously active GABAergic neurons targeted in Htr3a-Cre mice (i.e., SABIs) elicits temporally coincident barrages of large amplitude IPSCs in most SPNs. We hypothesize that the IPSCs originate from the disinhibition of a different population of striatal interneurons (not transduced in Htr3a-Cre mice) that are spontaneously active in the slice but whose activity is normally suppressed by the SABI. Candidates include the THINs and the LTS interneurons, but might also be another, as yet unidentified striatal GABAergic interneuron.

## Updates on Previously Identified Striatal GABAergic Interneurons

### Fast-Spiking Interneurons

#### Intrinsic Properties

As described previously, FSIs exhibit voltage-dependent membrane potential oscillations with peak power in the gamma range when depolarized. The oscillations become apparent during the characteristic long periods of silence that interrupt the depolarization induced intermittent firing of these interneurons in *ex vivo* whole cell recordings (Kawaguchi, 1993; Koós and Tepper, 1999). These oscillations are voltage-dependent, not blocked by the calcium channel blockers, nickel or cadmium, but are abolished by tetrodotoxin (TTX), indicating that they are Na^+^-dependent (Bracci et al., 2003).

In addition to these subthreshold oscillations, FSIs, like many other striatal interneurons, exhibit two types of resonance, a spiking resonance calculated by injecting an artificial synaptic barrage and measuring the phase of each spike relative to the peak of the injected current, and a subthreshold membrane resonance measured by a peak in the impedance amplitude spectrum (Beatty et al., 2015). The spiking resonance frequency for FSIs varied among different neurons, but was within the range of low to high gamma, and in some cases, a 2× higher harmonic. However, the membrane resonance frequency was significantly lower than the spiking resonance, around 20 Hz. Beatty et al. (2015) suggest that this may be due to the spiking resonance being due principally to axonal, not somatic resonance.

#### Subtypes of FSIs

Although the first descriptions of intracellular filling of electrophysiologically identified striatal FSIs suggested the possibility that there might be two morphologically distinct subtypes of FSIs (Kawaguchi, 1993; Koós and Tepper, 1999), the majority of studies, both *in vivo* and *ex vivo* have treated the PV immunoreactive FSIs as a singular cell population.

Recently, however, Garas et al. (2016) found that in both rat and primate (but oddly enough, not in mouse), FSIs could be separated into two populations depending on their expression of the calcium binding protein, secretagogin (Scgn). Furthermore, Scgn immunopositive (Scgn+) axons preferentially targeted the somata of direct pathway SPNs (dSPNs) whereas Scgn immunonegative (Scgn−) axons preferentially innervated indirect pathway SPNs (iSPNs), another striking example of the diversity of striatal GABAergic interneurons and the specificity of their inputs and outputs.

With subsequent *in vivo* extracellular recordings, these investigators identified these two subtypes of FSIs *post hoc* after juxtacellular biocytin labeling. First biocytin filled neurons were immunocytochemically identified as PV+ FSIs, and then these were subdivided by the presence or absence of immunoreactivity to Scgn. These studies revealed significant differences between Scgn+ and Scgn− FSIs in the relationship of their spiking to LFPs and the spiking of direct (dSPNs) and indirect pathway (iSPNs) projection neurons. Scgn− FSIs were more likely to precede firing of iSPNs than dSPNs while the converse was true for Scgn+ FSIs, consistent with the preferential innervation of iSPNs and dSPNs described above. Thus, the two subtypes of FSIs seem specialized to selective control feedforward inhibition of the direct and indirect pathways.

#### *In vivo* Activity and Behavior

A number of *in vivo* recording studies have characterized the spontaneous activity of putative FSIs, identified as such primarily on the basis of their very narrow extracellularly recorded action potential waveform (Berke et al., 2004; Berke, 2008, 2011). Presumed FSIs fired in a mostly random pattern while rats were awake, while exhibiting more complex bursty firing during slow wave sleep. Interestingly, even nearby presumed FSIs exhibited highly variable activity in response to cues and/or rewards, and there was little or no evidence for synchronous firing (Berke, 2008, 2011), as might be expected based on the demonstrated electrotonic coupling of FSIs *in vitro* (Koós and Tepper, 1999). While it is likely that most or all of these are FSIs, identification of neuronal type is much more problematic in *in vivo* extracellular recordings when based largely or exclusively on waveform and firing pattern than with *ex vivo* intracellular recording, biocytin labeling and immunocytochemistry.

#### FSIs and Behavior

A recent study confirms the importance of striatal FSI in mediating feedforward inhibition to SPNs after activation of corticostriatal input. In particular FSI would be important in the control of bursting of SPNs and in restricting plasticity, which facilitates sequence learning (Owen et al., 2018). Interestingly, FSIs show an elevated excitability in slices from mice trained in a habit formation behavior in comparison to goal-directed behavior. Consistently, acute chemogenetic inhibition of FSIs in dorsal lateral striatum prevents the expression of habitual lever pressing (O’Hare et al., 2017).

The role of striatal FSI in the learning of specific behavioral sequences was also recently investigated (Martiros et al., 2018). In this study the authors showed that SPNs and FSI have an opposite firing rate in relation to the completion of the behavioral sequence. While SPNs fired preferentially at the initiation and termination of the acquired sequence; FSIs fired in between the initiation and termination suggesting that SPN-FSI networks could underlie the acquisition of such behavior.

Striatal FSIs seem to be critical during early reward conditioning, while their importance in such behavior decreases with training and experience and the contribution of FSI on SPN activity consistently diminishes with training, suggesting that FSIs might act to enhance performance in the early stage of reward conditioning (Lee et al., 2017).

Selective inhibition of FSIs have been reported to produce dystonia-like movements in mice (Gittis et al., 2011), and differences in properties among putative FSIs in Genetically Hypertensive rats and Wistar rats suggest that FSIs may play a role in reducing impulsivity (Perk et al., 2015).

#### Afferents

In addition to cortical inputs, anatomical studies in primates have shown that FSIs receive thalamic inputs arising from thalamic intralaminar nuclei (centromedian-parafascicular nuclei). These thalamostriatal inputs seem to be significantly denser in primates than in rodents (e.g., Rudkin and Sadikot, 1999; Sidibé and Smith, 1999). Despite this, a recent comparison of responses to optogenetic stimulation of cortex and thalamus in mouse brain slices reveal very similar kinetics and amplitudes (Sciamanna et al., 2015). However, the corticostriatal synapses exhibited short term facilitation while the thalamostriatal synapses were depressing. One caveat is that the thalamic ChR2-YFP transduction in this latter study was not limited to the Cm-Pf, but appeared to involve most or all of the thalamus. It is thus significant that a very recent report showed a significant innervation of the proximal dendrites of PV-expressing FSIs arising from motor thalamus (Nakano et al., 2018) consistent with recent electrophysiological findings (Sciamanna et al., 2015; Assous et al., 2017; Arias-García et al., 2018; Assous and Tepper, 2018).

Although FSIs express both presynaptic muscarinic and postsynaptic nicotinic receptors (Koós and Tepper, 2002; Luo et al., 2013; Ibáñez-Sandoval et al., 2015), and there is electron microscopic evidence for cholinergic synapses on striatal PV neurons (Chang and Kita, 1992) there remains some confusion about the source of the cholinergic input. Neither electrical field stimulation (Koós and Tepper, 2002) nor optogenetic stimulation of striatal CINs (English et al., 2012; Assous et al., unpublished) is able to elicit any postsynaptic cholinergic response in FSIs. The reasons for this remain unclear. Perhaps the potent nicotinic response is volume conducted, or the cholinergic synapses that are observed in electron microscopic studies are inactivated in the *ex vivo* preparation for some unknown reason (Koós and Tepper, 2002). Lastly, it remains possible that the FSIs are innervated by recently described cholinergic afferents from the pedunculopontine nucleus (Dautan et al., 2014).

### LTS Interneurons

#### Intrinsic Electrophysiological Properties

In all of our previous publications, we (and many others) have used the original abbreviation coined by Kawaguchi (1993), PLTS, to refer to the striatal interneurons that coexpressed SOM, NPY and NOS due to the characteristic long lasting plateau potential often expressed by these neurons upon depolarization. However, recent article from the Wilson lab have revealed the plateau potential is an artifact of whole cell recording and is not seen in gramicidin perforated patch recordings (Beatty et al., 2012; Song et al., 2016), so these neurons are now more properly referred to as LTS interneurons.

Subthreshold membrane potential oscillations and membrane and spiking resonance have also been reported in LTS interneurons. Both spiking and membrane resonance were in the 10–30 Hz beta frequency range (Beatty et al., 2015). Unlike FSIs, the subthreshold oscillation is not Na^+^-dependent and in fact can be induced by TTX, although TTX-induced oscillations are slower (~4 Hz) than spontaneous oscillations occurring spontaneously (~8 Hz). Also in contrast to FSIs, the amplitude of the oscillations is dependent on Ca_v_1 and Ca_v_2.2 channels (Song et al., 2016). Most interesting, LTS interneurons were shown to express the calcium activated chloride channel (CaCC), ANO2 (as did NGF interneurons). Blocking these channels with the CaCC channel blocker, niflumic acid, abolished the oscillation and the membrane resonance, thereby demonstrating the same ionic mechanism for the subthreshold oscillations and the membrane resonance (Song et al., 2016).

#### Afferents

Anatomical and physiological data show that striatal LTS neurons receive a monosynaptic excitatory input from the cortex that can evoke both spikes and long lasting plateau potentials (Kawaguchi, 1993; Ibáñez-Sandoval et al., 2011; Assous et al., 2017; Choi et al., 2018). This contrasts with the cortical input to NGF interneurons discussed above. In addition, unlike NGF interneurons, FSI, CINs and THINs, LTS interneurons do not receive significant input from the PfN of the thalamus (Assous et al., 2017; Assous and Tepper, 2018). Indeed, almost half of the LTS interneurons do not respond to optogenetic stimulation of the PfN at all. The majority of those that do respond exhibit an IPSC instead of the expected EPSC. This IPSC has a longer latency than the EPSC in the NGF interneurons and can be blocked both by either bath application of GABA_A_ receptor or NMDA/AMPA receptor antagonists, thus indicating that the inhibitory response is polysynaptic. Using double transgenic mice and optogenetics we were able to show that the IPSC was at least in part mediated by monosynaptic thalamic glutamatergic activation of THINs that then make monosynaptic GABAergic synapses onto LTS interneurons (also see below).

Using retrograde tracing with modified rabies virus and slice recordings, a recent report highlights some discrepancies between anatomical and electrophysiological data. For example, similar to our study the vast majority of LTS interneurons do not respond to optogenetic stimulation of the PfN of the thalamus. However, the rabies anatomical tracing reveal dense innervation from this area of the brain (Choi et al., 2018). The reason for such discrepancies needs further investigation.

### TH Interneurons

#### Further Phenotypic Characterization

The most progress on the “new” striatal interneurons since 2010 has been made on striatal THINs. This is in part because both transgenic TH-EGFP reporter mice as well as TH-Cre mouse lines exist, and the neurons labeled in both strains are identical in all electrophysiological and morphological respects, which is sometimes not the case with other lines expressing either the reporter alone or the Cre-driver (e.g., the Htr3a-Cre (Faust et al., 2015) and the 5HT3A^EGF^ (Muñoz-Manchado et al., 2016).

Although in the original report describing THINs, they were shown to be GABAergic with paired whole cell recordings between THINs and SPNs (Ibáñez-Sandoval et al., 2010), the idea that they might also be dopaminergic neurons persisted on the basis of several indirect lines of evidence (e.g., Betarbet et al., 1997; Tandé et al., 2006; Huot and Parent, 2007; Darmopil et al., 2008; Ugrumov, 2009). Indeed, even some later reviews depicted them as dopaminergic interneurons (e.g., Gittis et al., 2010), despite the findings of Ibáñez-Sandoval et al. (2010) that these neurons made functional GABAergic synapses. This is not totally unreasonable since there exists many examples of neurons that can express and even release multiple neurotransmitters (for review see Tritsch et al., 2016), including dopamine and GABA (Tritsch et al., 2012).

Xenias et al. (2015) used TH-EGFP mice as well as TH-Cre mice and viral transduction to identify striatal THINs. Immunofluorescence controls performed in substantia nigra dopaminergic neurons identified in TH-EGFP mice revealed co-expression of dopamine and EGFP in most neurons. However, the same procedure failed to reveal any evidence of dopamine co-expression in THINs.

To determine why and how THINs express the gene for TH and the protein itself (albeit at very low levels, Ibáñez-Sandoval et al., 2010; Ünal et al., 2015) without also expressing dopamine, Xenias et al. (2015) tested THINs for colocalization of the dopamine transporter (DAT) and the vesicular monoamine transporter 2 (VMAT2), two enzymes necessary for uptake vesicular package of dopamine. Once again, positive controls performed in substantia nigra EGFP identified dopamine neurons revealed co-localization of both enzymes in substantia nigra dopamine neurons but not in any THINs. This explains how the THINs could express the TH gene and protein but not release dopamine.

As a final test, Xenias et al. (2015) performed *in vitro* voltammetry while optogenetically stimulating THINs in animals that were unilaterally depleted of dopamine by midbrain injection of 6-OHDA. On the control side, single 2.5 ms blue light stimuli elicited large (~1 μM) release of dopamine, but on the lesioned side, there was no measurable release of dopamine despite the fact that the optical stimulus caused spiking in simultaneously recorded THINs and GABAergic IPSPs and suppression of induced firing in SPNs. These data demonstrate conclusively that THINs are non-dopaminergic, GABAergic striatal interneurons that do not release dopamine, and further, that activation of THINs *in vivo* in experimental models of Parkinson’s disease does not activate “cooperative” synthesis and release of dopamine with other striatal interneurons, at least in brain slices, and likely *in vivo* as well, despite claims by others (Ugrumov, 2009; Kozina et al., 2017).

#### Afferents

THINs receive monosynaptic glutamatergic cortical inputs as they respond to cortical stimulation with EPSPs that elicit spiking, both of which are blocked by bath application of CNQX (Ibáñez-Sandoval et al., 2010). In addition, THINs are strongly innervated by the PfN of the thalamus where optogenetic stimulation elicits large depolarization and spiking in almost all recorded THINs (Assous et al., 2017).

Bath application of dopamine to THINs induces a dramatic enhancement of their characteristic long lasting plateau potentials in whole cell recordings after depolarizing somatic current injection (Ibáñez-Sandoval et al., 2015). This is consistent with anatomical evidence for the existence of dopaminergic varicosities closely apposed to THINs (Xenias et al., 2015). These data suggest that THINs might also be the target of midbrain dopaminergic neurons.

THINs also express nicotinic cholinergic receptors (Luo et al., 2013; Ibáñez-Sandoval et al., 2015) and are likely targets of striatal CINs and/or possibly afferents from the pedunculopontine nucleus (Dautan et al., 2014). Consistent with this receptor expression, optogenetic stimulation of striatal cholinergic axons (in ChATChR2 × THCre mouse) induce large depolarizations and action potential firing in recorded THINs (Assous et al., in preparartion; Assous and Tepper, 2018). Interestingly, unlike LTS, NGF and FS interneurons, the nicotinic responses of THINs are not blocked by Type II antagonists (Ibáñez-Sandoval et al., 2015) but are abolished by cytisine, a selective α_3_β_4_ Type III receptor antagonist (Luo et al., 2013), an example of pharmacological heterogeneity and diversity along with the previous examples of electrophysiological and functional heterogeneity and specificity.

#### Efferents

As mentioned above, evoked spiking in striatal THINs produces fast GABA_A_ IPSP/Cs in SPNs that are sufficient to significantly delay spikes evoked by intracellular depolarization. The IPSP/Cs are completely blocked by picrotoxin or bicuculline demonstrating that the THINs are GABAergic (Ibáñez-Sandoval et al., 2010). In addition we recently showed that THINs potently inhibit LTS interneurons (i.e., an interneuron to interneuron synapse), producing large IPSP/Cs that are able to induce a pause in the firing of spontaneously active LTS interneurons. This direct monosynaptic connection is responsible for the disynaptic inhibition of LTS interneurons after optogenetic stimulation of the PfN of the thalamus, resulting in opposite effects of thalamic input onto the two types of striatal NPY interneurons (Assous et al., 2017).

Our preliminary data also suggest a direct connection between THINs and CINs (Assous and Tepper, 2018; Assous et al., unpublished). It is interesting to note that in contrast to LTS interneurons and CINs, THINs do not innervate FSI or NGF interneurons (Assous et al., 2017), providing another example of the complexity and specificity of the intrastriatal interneuronal networks (Straub et al., 2016).

#### Calretinin Interneurons

CR interneurons (together with FSI and LTS interneurons) are one of the three first, classically identified GABAergic interneurons in the rodent striatum, characterized largely on the basis of immunocytochemistry. Unlike the FSIs and the LTS interneurons, along with the more recently described THINs, NGF interneurons, FAIs and SABIs, we still know very little about striatal CR interneurons due to the lack of any transgenic Cre-driver or fluorescent reporter mice that works reliably in striatum, much as was the case in 2010.

However, very recently, the first *in vivo* recordings and juxtacellular labeling of CR interneurons identified *post hoc* by immunocytochemistry were reported (Garas et al., 2017). The authors report that there are actually three structurally and topographically distinct CR population as we suggested based on the differential anatomy of immunostained CR interneurons in our last review (Tepper et al., 2010). Garas et al. (2017) showed that the subtypes of CR populations could be defined based on the combinatorial expression of Scgn, specificity protein 8 and/or LIM homeobox protein 7 (Lhx7). *In vivo* recordings in anesthetized rats of one of the subtypes of CR, reveal that these neurons (CR+, Scgn−, Lhx7−) have a very variable firing pattern during cortical slow wave activity. During cortical activation, all recorded CR interneurons displayed tonic activity (Garas et al., 2017).

Interestingly, in primates it also seems that three types of CR exist based on soma size and morphologies (Petryszyn et al., 2016). In this study the authors also report that the “large” CR interneurons also express ChAT, a difference between primates and rodents.

### Functional Significance

In structures such as the cortex and hippocampus, which are often used as model structures, interneurons make up about 15%–30% of the neuronal populations and are incredibly diverse with 10–15 different interneuron subtypes depending on the classification method. Given the size and importance of the striatum to basal ganglia function, it is not very surprising that the diversity of striatal interneurons is far greater than originally thought. As reviewed above, in addition to the three classically described striatal GABAergic interneurons (PV, LTS, CR) we now know of the existence of at least four additional cell types (THINs, NGF, FAI and SABIs); and it is likely that we have not yet found all of them; at least the interneuron responsible for recurrent inhibition of striatal CINs and the interneuron(s) responsible for the disinhibitory IPSCs measured in SPNs after inhibition of Htr3a targeted interneurons remain unknown.

Of equal importance, our knowledge of the striatal interneuronal circuitry has greatly expanded. We know about fast excitatory synaptic interactions mediated by nicotinic synapses between CINs (and possibly brainstem cholinergic afferents) and GABAergic interneurons that provide a third source of potent excitation in addition to the cortical and thalamic glutamatergic inputs. And there is now evidence for some significant differences between cortical and thalamic inputs to different GABAergic interneurons (Assous and Tepper, 2018). Adding in the existence of homo- and heterotypic electronic connections among many different subtypes of GABAergic interneurons, and the first evidence for interneuron selective interneurons, the intrastriatal circuitry is considerably more complex than it was in 2010.

All striatal interneurons tested so far (and that is only a minority of the total) exhibit different “best” or spiking resonance frequencies (Beatty et al., 2015; Song et al., 2016). One must layer on top of all of the considerations discussed above that different subtypes of striatal GABAergic interneurons are “tuned” by these resonances to different frequencies of afferent input. Thus, the same excitatory cortico- and thalamostriatal synaptic inputs will have specific and selective effects on different subtypes of GABAergic interneurons, depending on moment to moment changes in firing rates of the afferent inputs (Beatty et al., 2015).

The existence of large-scale ensembles of SPNs acting as functional units in striatum has been hypothesized for a long time (e.g., Jog et al., 1999; Carrillo-Reid et al., 2008). Such ensembles have been assumed to be formed principally by convergent excitatory inputs and Hebbian plasticity in cortical and hippocampal regions where the principal cells forming the ensembles are excitatory. This is not the case in striatum, where the SPNs are GABAergic and inhibit one another. However, it is clear that local inhibitory inputs exert significant modulatory effects on hippocampal neuronal ensembles (e.g., Stefanelli et al., 2016) as well as in striatal neuronal ensembles (Lee et al., 2017).

The data reviewed reveal highly selective and specific synaptic connections between different interneuron subtypes and SPNs and also among different GABAergic interneurons themselves, suggesting the existence of a hierarchical control of other striatal interneurons. We suggest that this highly organized striatal circuitry is a major factor in the formation and maintenance of functional networks and ensembles of SPNs.

## Author Contributions

JT, TK and MA designed some of the experiments reviewed. OI-S, FT, TK, TF and MA performed some of the experiments reviewed. JT and MA wrote the manuscript.

## Conflict of Interest Statement

The authors declare that the research was conducted in the absence of any commercial or financial relationships that could be construed as a potential conflict of interest.

## Abbreviations

CaCC: calcium activated chloride channel
ChAT: choline acetyltransferase
CIN: cholinergic interneuron
CR: calretinin
DAT: dopamine transporter
dSPN: direct pathway spiny projection neuron
FAI: fast adapting interneuron
FSI: fast spiking interneuron
iSPN: indirect pathway spiny projection neuron
Lhx7: lIM homeobox protein 7
LTS: low threshold spike
NGF: neurogliaform
NOS: nitric oxide synthase
NPY: neuropeptide Y
PfN: parafascicular nucleus of the thalamus
PV: parvalbumin
RMP: resting membrane potential
SABI: spontaneously active bursty interneuron
Scgn: secretagogin
SOM: somatostatin
TH: tyrosine hydroxylase
TTX: tetrodotoxin
VMAT2: vesicular monoamine transporter 2.
